# A new cell culture model to genetically dissect the complete human papillomavirus life cycle

**DOI:** 10.1371/journal.ppat.1006846

**Published:** 2018-03-01

**Authors:** Malgorzata Bienkowska-Haba, Wioleta Luszczek, Julia E. Myers, Timothy R. Keiffer, Stephen DiGiuseppe, Paula Polk, Jason M. Bodily, Rona S. Scott, Martin Sapp

**Affiliations:** 1 Department of Microbiology and Immunology, Center for Molecular and Tumor Virology, Feist-Weiller Cancer Center, Louisiana State University Health Sciences Center, Shreveport, Louisiana, United States of America; 2 Research Core Facility, Louisiana State University Health Sciences Center, Shreveport, Louisiana, United States of America; University of Wisconsin Madison School of Medicine and Public Health, UNITED STATES

## Abstract

Herein, we describe a novel infection model that achieves highly efficient infection of primary keratinocytes with human papillomavirus type 16 (HPV16). This cell culture model does not depend on immortalization and is amenable to extensive genetic analyses. In monolayer cell culture, the early but not late promoter was active and yielded a spliced viral transcript pattern similar to HPV16-immortalized keratinocytes. However, relative levels of the E8^E2 transcript increased over time post infection suggesting the expression of this viral repressor is regulated independently of other early proteins and that it may be important for the shift from the establishment to the maintenance phase of the viral life cycle. Both the early and the late promoter were strongly activated when infected cells were subjected to differentiation by growth in methylcellulose. When grown as organotypic raft cultures, HPV16-infected cells expressed late E1^E4 and L1 proteins and replication foci were detected, suggesting that they supported the completion of the viral life cycle. As a proof of principle that the infection system may be used for genetic dissection of viral factors, we analyzed E1, E6 and E7 translation termination linker mutant virus for establishment of infection and genome maintenance. E1 but not E6 and E7 was essential to establish infection. Furthermore, E6 but not E7 was required for episomal genome maintenance. Primary keratinocytes infected with wild type HPV16 immortalized, whereas keratinocytes infected with E6 and E7 knockout virus began to senesce 25 to 35 days post infection. The novel infection model provides a powerful genetic tool to study the role of viral proteins throughout the viral life cycle but especially for immediate early events and enables us to compare low- and high-risk HPV types in the context of infection.

## Author summary

Current cell culture models for the study of the human papillomavirus (HPV) life cycle depend on immortalized keratinocytes harboring episomal HPV genomes. However, the requirement for immortalization restricts the study to only a few HPV types and does not allow investigating immediate early events of the viral life cycle. Despite many efforts, efficient infection of primary keratinocytes has not been achieved until now. Using pre-binding of virus to extracellular matrix deposited by keratinocytes, we now achieve very efficient infection of primary keratinocytes. The infection model allows studying the complete viral lifecycle. It could be extended to HPV types that do not immortalize keratinocytes and allows an extensive genetic screen of the contributions of viral factors throughout the viral lifecycle. It should aid the investigations of processes leading to HPV-induced immortalization.

## Introduction

High-risk HPV types such as HPV16 are the infectious agents most commonly associated with human cancers such as but not restricted to cervical and oropharyngeal squamous cell carcinoma. Approximately 5% of all human cancers can be linked to HPV infection. HPV encodes two major viral oncoproteins, E6 and E7, which drive immortalization and transformation of HPV infected cells. Their roles in cancer development can be mostly attributed to the inactivation of the p53 [1–3] and pRb family of tumor suppressors [4], respectively. The viral oncogenes have been extensively studied over the past three decades mainly using transfection models and recombinant retroviruses to express them in established and primary keratinocytes.

However, immortalization and transformation are not the default outcome of an HPV infection. Instead, oncogene expression is tightly regulated in a natural infection. Our understanding of this regulation is very limited. The lack of knowledge is partly due to the fact that the HPV life cycle is strictly dependent on the terminal differentiation process of keratinocytes making the studies technically difficult. Our current view is that HPV gains access to stem and post stem cells of the basal layer through (micro)lesions by preferentially binding to the basement membrane (BM) [5]. After reaching the nucleus, it is assumed that viral genome is initially amplified. This is based on the observation that up to several hundred copies of viral genome can be found in infected basal keratinocytes [6]. After establishment of infection, the viral genome copy number is maintained in the basal compartment by maintenance replication. Viral transcription occurs at a low rate and it is assumed that the infection spreads by cell division. When HPV-harboring keratinocytes enter the terminal differentiation program, viral transcription is activated [7]. Uninfected keratinocytes exit the cell cycle at this time and commit to terminal differentiation. However E7 protein, which negates the function of the pRb family members, allows HPV-harboring cells to maintain cell cycle competence. As a consequence, E1 and E2 protein in concert with the host cell replication machinery amplify the viral genome [8]; a process that requires activation of the DNA damage response [9] and the function of the E4 and E5 viral proteins through poorly understood mechanisms [10, 11]. Inactivation of p53 by E6 protein prevents cell cycle arrest due to unscheduled DNA replication. The viral life cycle is completed following structural (late) gene expression and assembly of progeny virions in highly differentiated cells of the uppermost layers of the stratified epithelium [12].

Most of our current knowledge is based on studying HPV-harboring keratinocytes either derived from lesions or established after transfection of the viral genome. However, establishment of these cell lines requires outgrowth of immortalized keratinocytes, which in turn depends on viral oncogene expression. According to current models, immortalization is associated with increased expression of E6 and E7 [13]. Therefore, HPV-harboring cells likely display deregulated viral oncogene expression and may not be suitable for the investigation of viral early promoter regulation after infectious entry. Thus, essentially no information is available regarding the early events that regulate viral oncogene expression in an HPV-infected basal cell; despite our detailed understanding of processes leading to tumor progression. Similarly, many assumptions about establishment of infection and shift to maintenance such as genome amplification during the establishment phase lack robust experimental support. This lack of knowledge can be attributed to the fact that no cell culture model has been available to study the immediate early events of the HPV life cycle, despite more than 20 years of effort by many researchers in the field. While significant recent advances have allowed generation of virions using packaging cell lines or organotypic raft cultures [14–18], we have been unable to infect primary keratinocytes efficiently for the study of the complete viral life cycle. Even though two reports published in 2009 described efficient infection of primary keratinocytes with HPV18, the system does not seem to be robust as no follow up studies were reported [19, 20].

We have now succeeded in developing an infection model that mimics immediate early events of the HPV life cycle. The infection model is amenable to extensive genetic screens, could possibly be expanded to essentially all HPV types and allows the completion of the viral life cycle. This represents a significant technological advance that will enable the HPV and cancer research community to fill in huge gaps in our understanding of the regulation of oncogene expression and its deregulation in the early stages of tumor development. Our model will also be extremely helpful in gaining a better understanding of the HPV life cycle. It should allow a direct comparison of high- and low-risk HPV types for the first time.

## Results

### Efficient HPV16 infection of primary keratinocytes after ECM-to-cell transfer

**Fig 1:**
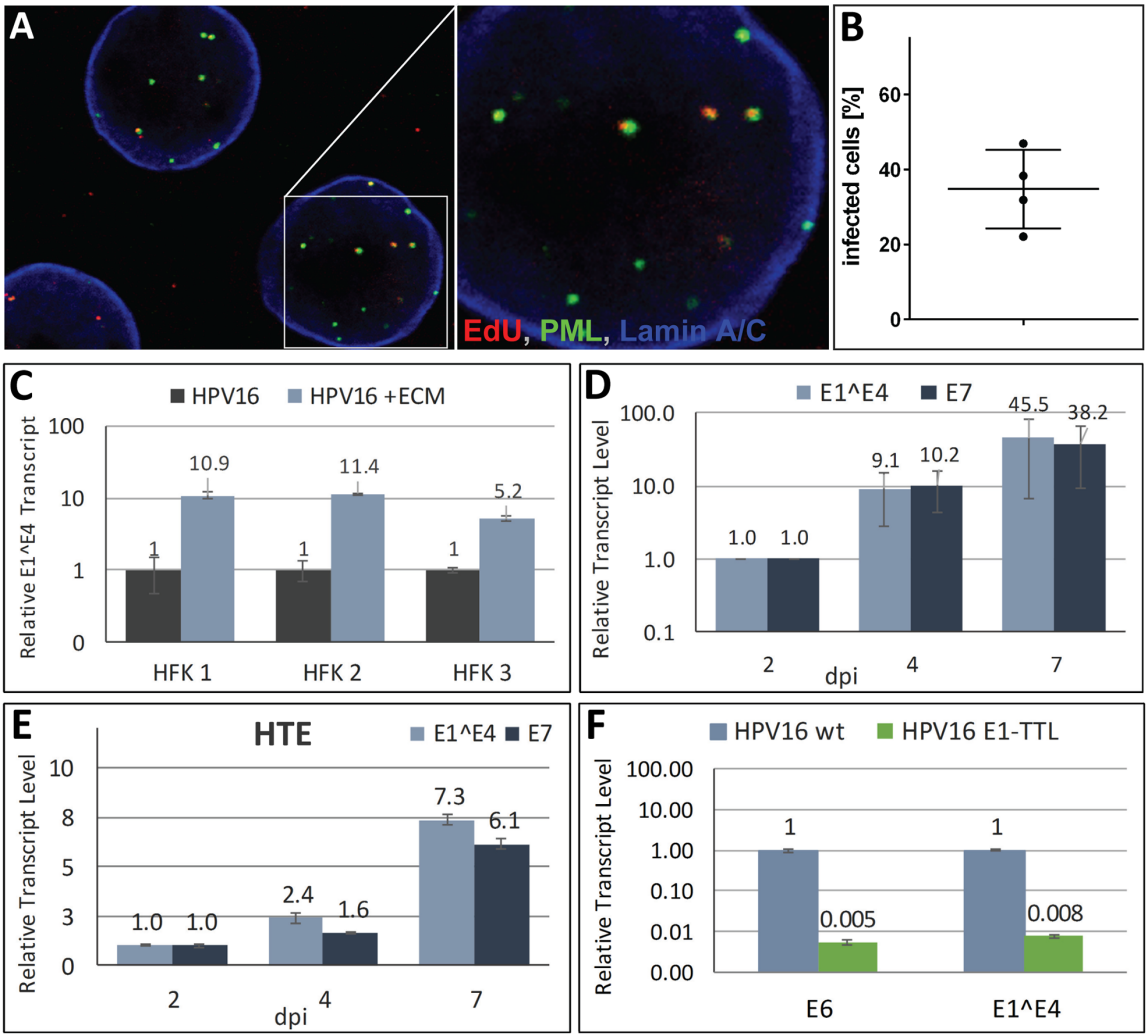
Efficient infection of primary keratinocytes. **(A)** HFK cells were infected with EdU-labeled HPV16 pseudovirus using ECM-to-cell transfer. At 40 hpi, cells were fixed and processed for the detection of EdU-labeled DNA (red), PML (green), lamin A/C (blue). A representative image of HFK cells infected with EdU-labeled pseudovirus is shown. **(B)** Quantification of HFK cells from two different donors containing nuclear EdU-labeled pseudogenome at 40hpi. EdU-labeled viral pseudogenome number was counted manually in z-stacks spanning the whole nucleus for each cell as expressed as percent of analyzed cells. 72 & 60 and 64 & 77 cells were analyzed for HFK1 and HFK2, respectively. **(C)** HFKs from three different donors were infected with HPV16 via ECM-to-cell transfer or by direct binding to cells. At 72 hpi, RNA was isolated and E1^E4 transcript was measured by RT-qPCR. The data are presented as fold changes relative to cells infected by direct virion binding. **(D and E)** E7 and E1^E4 transcript levels increase with prolonged exposure to virus-loaded ECM in primary HFK (D) and HTE (E). **(F)** RT-qPCR analysis of viral transcripts at 6 dpi of HFK with wt and E1-TTL mutant HPV16 virus. Unless otherwise noted, all results are based on three biological replicates. HFK were maintained and infected in the presence of 10 μM Y-27632 for experiments shown in panels A, B, D, and F.

Direct binding of HPV16 to primary keratinocytes yields very inefficient infection rates for unknown reasons. However, it was reported that HPV16 preferentially binds *in vivo* and *in vitro* to the basement membrane and the extracellular matrix (ECM) secreted by keratinocytes, respectively [21–23]. The interactions with ECM-resident receptors such as LN332 and heparan sulfates were shown to be sufficient to induce conformational changes in viral capsid proteins that are important for infectious entry. Mutational analyses of receptor binding sites also suggested a unique contribution of LN332 to conformational shifts in capsid proteins [24–26]. Based on these findings, we hypothesized that pre-binding of virions to ECM depositions would mimic *in vivo* infection and improve infection of primary keratinocytes. To test this, HaCaT cells were grown in culture dishes for 48 h and subsequently removed by treatment with EDTA. Next, HPV16 viral particles generated using the 293TT packaging cell line were added to the ECM depositions left behind on the culture dish, incubated for 2 h and followed by seeding of primary keratinocytes. With this protocol, we were able to deliver EdU-labeled pseudogenome to the nuclei of close to 50% of primary human foreskin keratinocytes (HFK) at 40 hours post infection (hpi) using ECM-to-cell transfer (Fig 1A and 1B). We observed that HPV16 E1^E4 transcripts were 10-fold higher following ECM-to-cell transfer of HPV16 virions as compared to direct binding to HFK at 72 hpi (Fig 1C). E7 and E1^E4 transcript levels were further increased up to 50-fold when HFK were left on the ECM for 7 instead of 2 days with transcripts arising mostly from the early promoter (Fig 1D). Detaching infected cells from virus-loaded ECM at day 2 and reseeding on ECM-coated dishes did not yield higher transcript levels (S1A Fig). This finding suggests that increased transcript levels over time can be attributed to the continual delivery of viral genome rather than increased promoter activity. We were also able to efficiently infect primary human tonsilar epithelial (HTE) cells using this ECM-to-cell transfer to deliver viral genome (Fig 1E). HeLa cell secretions, which lack LN332, do not support efficient HPV16 infection (S1B Fig). This is in line with previous observations, which suggested that ECM-resident LN332 plays an important role in efficient ECM-to-cell transfer [22]. HPV16 virions harboring a translation termination linker (TTL) mutation in E1 failed to establish infection since viral transcripts were hardly detectable (Fig 1F) suggesting that E1 is essential for establishment of HPV16 infection and providing indirect support for the amplification of incoming viral genome.

**Fig 2:**
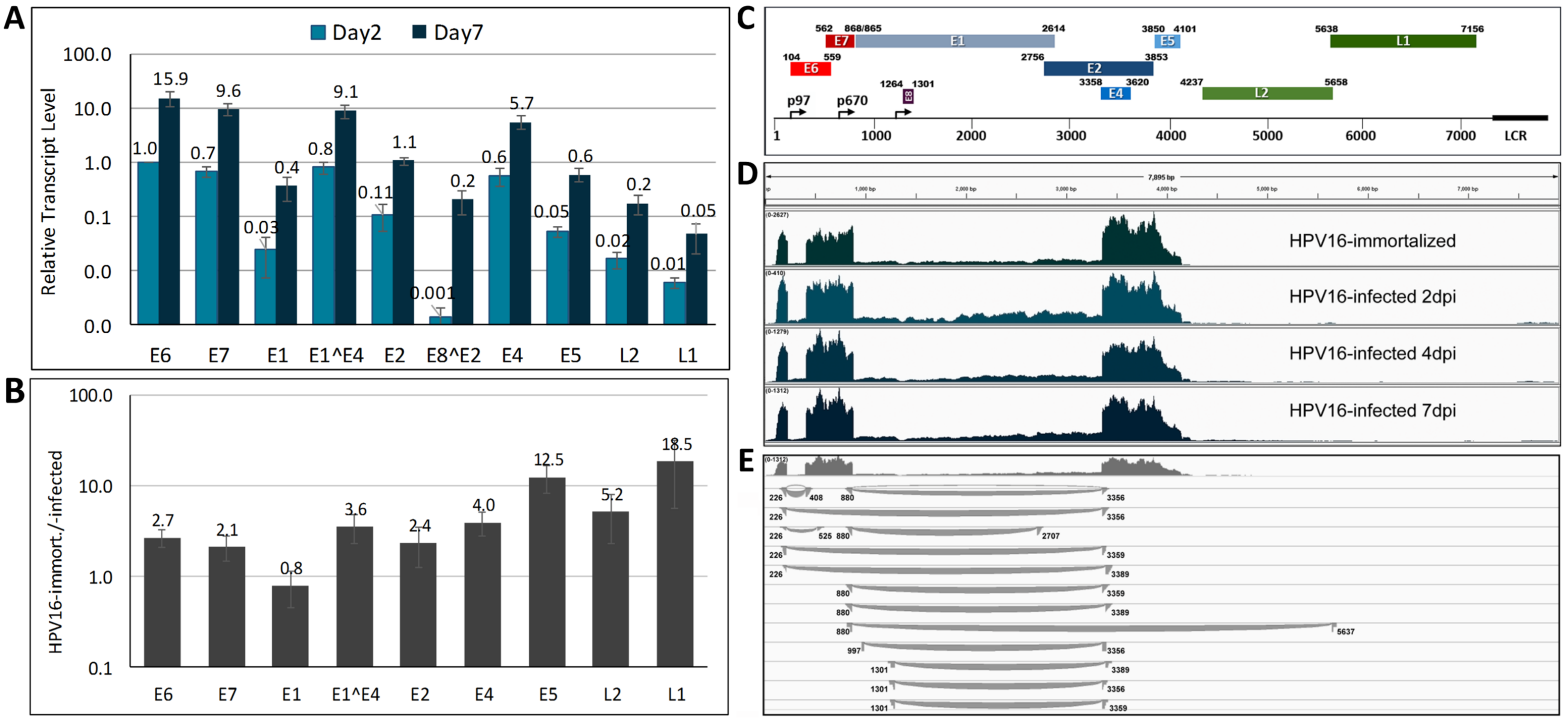
Analysis of viral transcripts by next generation RNA sequencing. **(A)** Relative expression levels of individual viral ORFs in HPV16-infected HFK at 2 and 7 dpi. Cells grown in the presence of 10 μM Y-27632 were infected with HPV16 quasivirions via ECM-to-cell transfer and transcripts were quantified by RT-qPCR. The data shown are fold changes normalized to E6 transcript levels at 2 dpi of HFK. **(B)** RT-qPCR analysis of viral transcripts isolated from HPV16-immortalized HFKs and normalized to transcript levels in HPV16-infected HFKs at 10 dpi. **(C)** Schematic representation of the HPV16 genome and its ORFs. **(D)** Read depth maps of viral transcripts isolated from HPV16-infected HFK at 2, 4 and 7 dpi and from HPV-immortalized HFK of representative samples. **(E)** Detailed analysis of splice junctions of viral transcripts isolated from HFK at 7dpi. Each curved line represents a splice junction derived from individual reads that connects splice donor and acceptor sites.

HPV16 early transcripts are transcribed from the early promoter p97 and are differentially spliced resulting in different quantities of viral open reading frames (ORF) (for a review see [27]). The late promoter p670 is activated when infected keratinocytes enter the terminal differentiation program. As expected when primarily the p97 early promoter is active, the most abundant transcripts contained the E6, E7 and E4 ORFs, whereas the early E1, E5 and E2 transcripts were present at significantly lower levels (Fig 2A). The late L1 and L2 ORFs were essentially undetectable at two days post infection (dpi) of HFK and just barely reached our limit of detection at 7 dpi suggesting that the late promoter is under tight control in infected HFK. Similar results were obtained with HPV16-infected HTE (S2 Fig). When we compared viral transcript levels between HPV16-infected and -immortalized HFK, we found that most early transcripts were present at 2- to 4-fold lower levels in HPV16-infected HFK (Fig 2B), with the exception of E1 encoding transcripts for which we found similar levels. The transcripts containing the late L1 and L2 ORFs as well as the E5 ORF were found at up to 20-fold lower levels in HPV16-infected compared to -immortalized HFK. The data imply a very tight control of the late promoter after HPV infection.

We profiled RNA derived from HPV16-infected HFK at 2, 4 and 7 dpi using next generation sequencing (NGS) and compared the outcome to RNA isolated from HPV16-immortalized HFK. The overall profile of the viral transcripts isolated from HPV16-infected and -immortalized HFK is very similar despite differences in read depths, providing further support for the validity of the infection model (Fig 2C and 2D). Two major splicing events use the 226 and the 409 (E6*I) and the 880 and 3358 (E1^E4) splice acceptor and donor sites, respectively. Approximately 40 to 45% of all early transcripts are spliced at the 226/409, 40 to 43% use the 880/3358 splice donor and acceptor pair. Additional previously described junctions 226/526 (E6*II; 2.5–3.1%), 226/3358 (3–5.8%), 880/2709 (E2; 3.1–3.8%), and 880/3391 (2.4–3.1%), were also found at lower frequency (Fig 2D and 2E; S1 Table). In addition to the splice acceptor site at 3358, an alternative site at 3361 is being used at low frequency. The splice variant with E8^E2 coding potential (1302/3358) is the only one, whose relative levels increase significantly over time post infection compared to other early transcripts (S1 Table) suggesting that it may be important for a switch to maintenance replication and offering support for previous reports suggesting a repressive role for E8^E2 [28–31]. Less than 5% of the early transcripts have coding potential for full-length E6. The NGS results also confirm the low abundance of E1 and E2 encoding RNAs (Fig 2A). Some minor splice variants previously reported in HPV16-immortalized cells and confirmed by our analysis were not present in HPV16-infected cells (S1 Table).

### The early and late promoters are activated by differentiation

**Fig 3:**
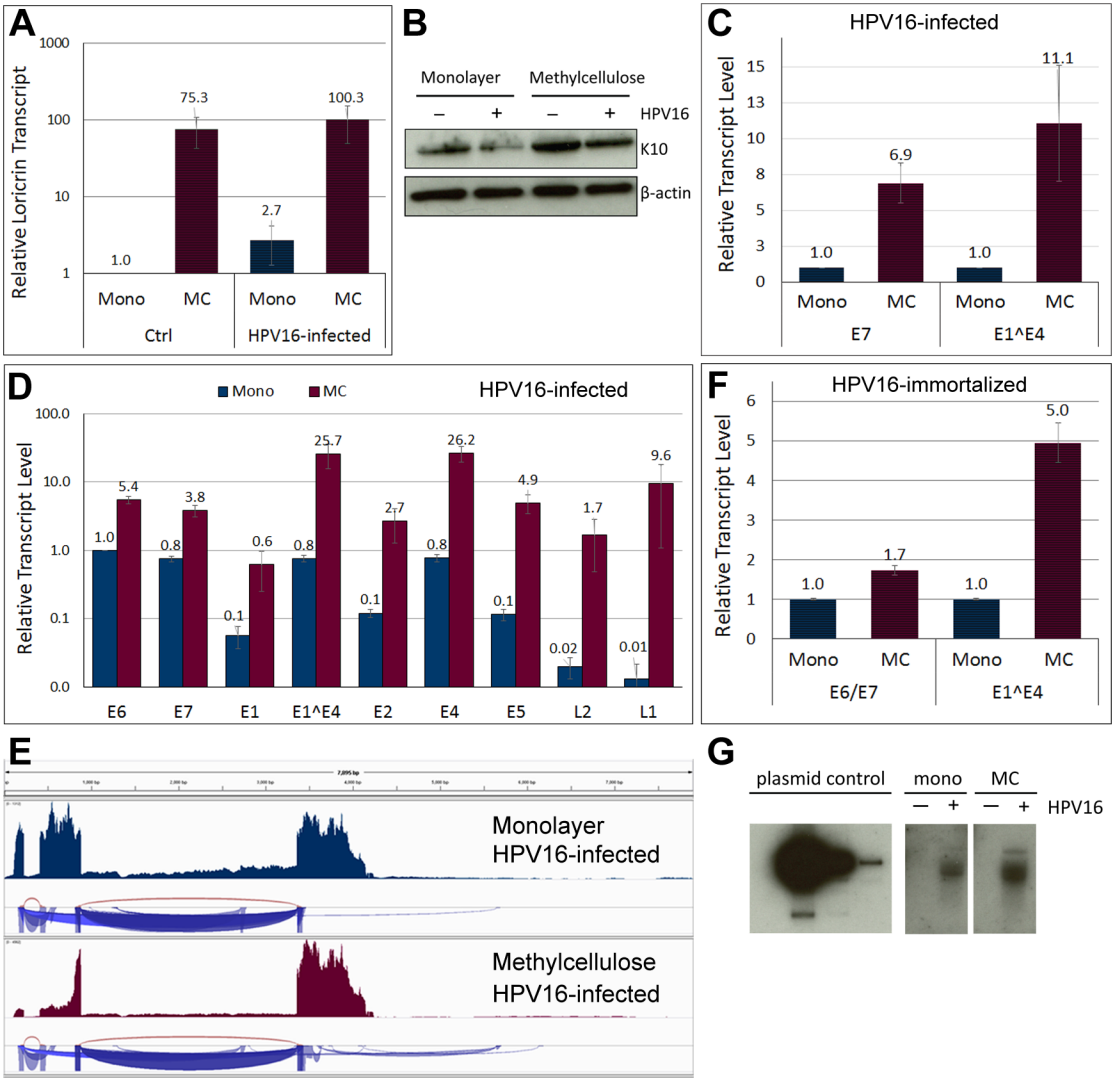
Differentiation activates both viral promoters and results in genome amplification. HFKs maintained and infected in the presence of 10 μM Y-27632 for 5 days with HPV16 were subjected for 24–48 h to differentiation by plating in MC. **(A)** RT-qPCR analysis of loricrin transcript level in cells grown in monolayer and MC. The data shown are fold changes relative to uninfected HFK cells grown in monolayer. Error bars represent SEM of three independent experiments. **(B)** Western blot for keratin 10 in cells grown in monolayer and MC. **(C)** Expression of E7 and E1^E4 in HPV16 infected HFKs. The data shown are fold changes relative to cells grown in monolayer. Error bars represent SEM of three independent experiments. **(D)** Relative expression levels of individual ORFs in differentiated HPV16 infected cells. The data shown are fold changes normalized to E6 transcript levels in infected cells grown in monolayer. Error bars represent SEM of four biological replicates. **(E)** Read depths maps of viral transcripts isolated from HPV16-infected HFK grown in monolayer and MC. **(F)** Expression of E6/E7 and E1^E4 in HPV16 immortalized HFKs. The data shown are fold changes relative to cells grown in monolayer. Error bars represent SEM of six independent experiments. **(G)** Southern blot for viral genome isolated from HPV16-infected HFK grown in monolayer and MC.

To test whether the incoming viral HPV16 genome is responsive to differentiation, we subjected HFK infected for 5 days with HPV16 virions to growth in semi-solid methylcellulose (MC) media, which is well established to induce differentiation of keratinocytes and to activate the viral late promoter [32]. Differentiation was confirmed by increased expression of differentiation markers loricrin and keratin 10 by RT-qPCR (Fig 3A) and by Western blot (Fig 3B), respectively. Activation of the late promoter was observed by RT-qPCR and confirmed by NGS giving rise to late L1- and L2-encoding transcripts (Fig 3C–3E). In addition, the early promoter was activated as evidenced by a 7-fold increase of early transcripts (Fig 3C). This was seen when HFK were grown in the presence and absence of the ROCK inhibitor. We would like to point out that the E1^E4 transcript measured in Fig 3C can arise from both the early and late promoter. In contrast, growth of HPV16-immortalized HFK in MC activated the late but only weakly the early promoter (Fig 3F). Southern blot analysis of viral genome also suggested increased viral genome levels after growth of HPV16-infected HFK in MC (Fig 3G). These data indicate that the viral genome delivered by HPV16 particles establishes infection and responds to differentiation. Furthermore, our data suggest that not only the late but also the early promoter responds to differentiation, thus providing the first experimental evidence of what has been previously implied, albeit indirectly, by RNA *in situ* hybridization of naturally infected lesions [33, 34].

### Organotypic raft culture of HPV16-infected HFK

**Fig 4:**
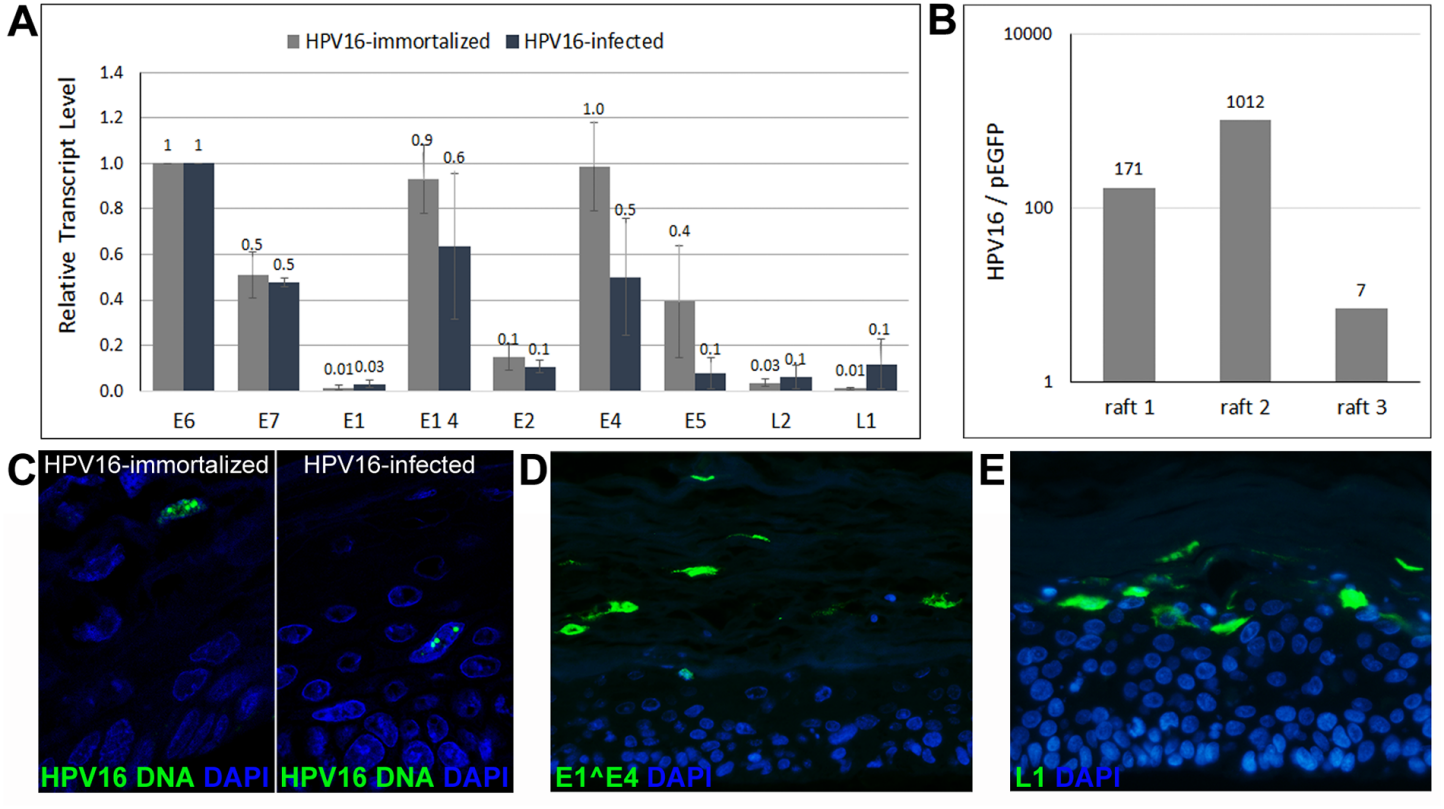
Genome amplification and capsid protein expression in HPV16-infected HFK subjected to organotypic raft culture. **(A)** Transcription profile of viral transcripts from rafts derived from HPV16-immortalized and -infected HFK. The data shown are fold changes normalized to E6 transcript levels. Error bars represent SEM of three independent experiments. **(B)** HPV16 genome is enriched in organotypic rafts over pEGFPN1 plasmid. **(C)** Thin cuts of organotypic rafts generated from HPV16-immortalized and -infected HFK cells subjected to HPV16-specific FISH. **(D and E)** E1^E4 (D) and L1 (E) protein are expressed in rafts grown from HPV16-infected HFK. Nuclei were stained with Dapi.

**Fig 5:**
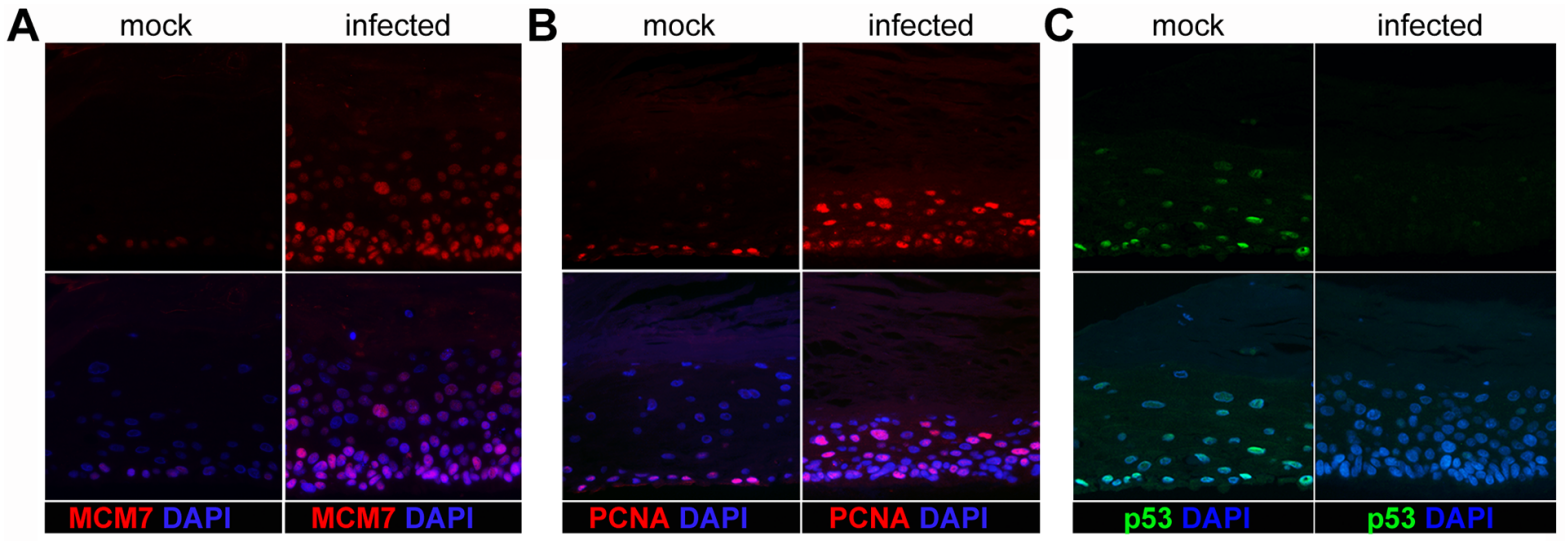
Immunofluorescent detection of cellular markers in organotypic rafts grown from HPV16-infected HFK. Thin cuts of organotypic rafts were stained for MCM7 **(A)**, PCNA **(B)**, and p53 **(C)**. The lower panels show a merge with the Dapi stain to highlight nuclei.

We next subjected HFK infected for 5–7 days with HPV16 to organotypic raft cultures, which have previously been shown to support completion of the viral life cycle [18]. Uninfected and HPV16-immortalized HFK served as negative and positive controls, respectively. As shown in Fig 4A, both early and late transcripts were detectable in rafts and the expression profile of viral RNA isolated from rafts derived from infected and immortalized HFK were similar, albeit total viral RNA levels tended to be lower in rafts from HPV16-infected cells. We also observed that HPV16 genome was retained in the raft cultures, thereby suggesting replication of viral genome has occurred (Fig 4B). Indeed, HPV16-specific fluorescent *in situ* hybridization (FISH) identified cells with replication foci in rafts derived from both HPV16-immortalized and -infected HFK (Fig 4C). Immunofluorescent staining for E1^E4 and L1 protein were positive in many cells of the upper layers of the raft tissues (Fig 4D and 4E). Furthermore, markers of cell proliferation such as MCM7 and PCNA were present throughout the parabasal and spinous layers of the stratified epithelia and p53 signal was greatly diminished in HPV16-infected but not mock-infected cells (Fig 5). These results confirm our previous observation that most cells had been infected. Taken together, amplification of the viral genome and the presence of L1 protein suggest that the ECM-to-cell transfer infection model allows recapitulation of the complete viral life cycle.

### E6 but not E7 is essential for genome maintenance in monolayer cell cultures

**Fig 6:**
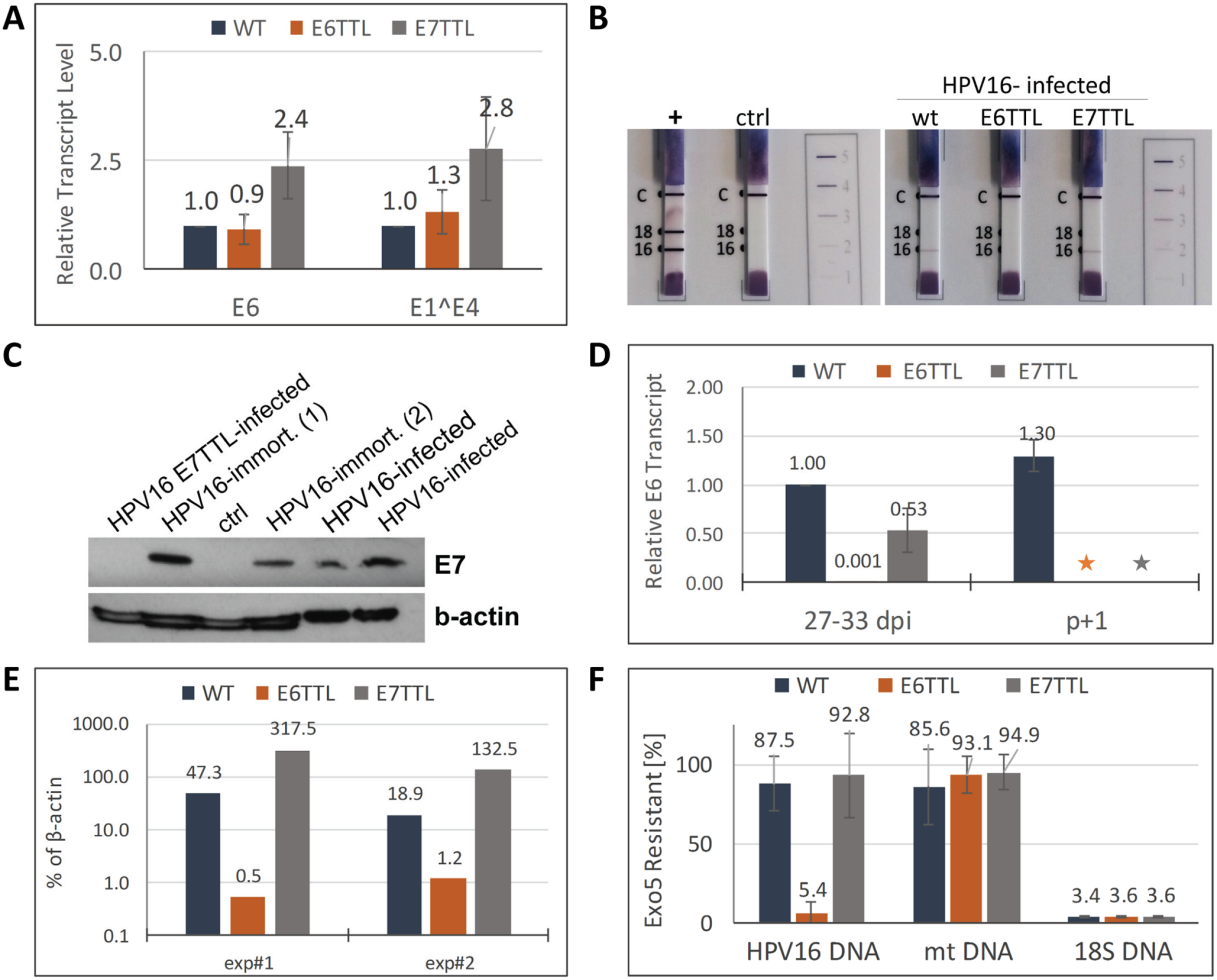
E6 but not E7 is required for episomal genome maintenance in monolayer cells. **(A)** E6 and E1^E4 transcript levels at 6 dpi of HFK with wt, E6-, and E7-TTL mutant virus. **(B)** E6 protein detection using the ArborVita Onco*E6*^*™*^ Cervical Test. The test is described in detail in Material and Methods. Note the absence of the E6-specific band in samples derived from control and E6-TTL mutant virus infected HFK. **(C)** E7 protein detection by western blot. Note the absence of E7 protein in samples derived from HFK infected with E7-TTL mutant virus. Samples from two different HPV16-immortalized HFK lines were analyzed. Ctrl: extracts from mock-infected HFK. **(D)** E6 transcript levels at 27 to 33 dpi with the indicated virus. The samples were taken at the last passage before control, E6-, and E7-TTL virus infected HFK underwent senescence. We also obtained samples from HFK infected with wt HPV16, which were the only cells to become immortalized, one passage later. *: indicates that these cells had senesced. **(E)** Quantification of viral genome by qPCR isolated from HFK at 27–33 dpi with respective virus and expressed as percent of ß-actin DNA levels. **(F)** Viral genome remains episomal in HFK infected with wt and E7-TTL but not with E6-TTL virus. DNA isolated from HFK infected with respective virus was subjected to exonuclease 5 treatment. Resistant DNA was quantified by qPCR. Primers for amplification of HPV16, mitochondrial (mt) DNA and 18S DNA were used.

As proof of principle that the infection model is amenable to genetic analyses, we generated HPV16 mutant viruses harboring translation termination linkers in the E6 and E7 open reading frames. Both mutant viruses established infection as evidenced by the presence of early transcripts (Fig 6A). We subjected extracts derived from HFK infected with respective wild type (wt) and mutant virus at 7 dpi to western blot analysis and a commercially available test for detection of E7 and E6 protein, respectively. E6 and E7 proteins were detected in HFK infected with wt HPV16 but were absent after infection with the respective mutant virus (Fig 6B and 6C). We conclude that expression of E6 is not impaired by E7 knockout and vice versa.

We also subjected HFK infected with mutant and wt HPV16 to long-term culturing to monitor cell survival, viral transcript, and genome levels. We observed almost complete loss of viral transcripts within 27–33 dpi with E6-TTL mutant virus (Fig 6D). This was accompanied by a loss of viral genome (Fig 6E). In contrast, HFKs infected with the E7-TTL mutant retained high levels of viral transcripts (Fig 6D). To test whether viral genomes were maintained as episomes, we developed an assay determining the resistance of HPV16 genome to exonuclease 5. Intact double-stranded circular DNA is not a substrate for this enzyme. DNA was isolated from HFK infected with wt, E6-, and E7-TTL mutant virus at 29–33 dpi, treated with exonuclease 5 and subjected to qPCR. 18S ribosomal DNA was completely digested in all samples indeed confirming that the nuclease treatment was sufficient for removal of linear DNA (Fig 6F). In contrast, mitochondrial DNA was mostly resistant as expected for a circular DNA molecule. We found that HPV DNA isolated from cells infected with wt and E7-TTL mutant virus was mostly resistant confirming that they are not substrates for exonucleases and thus likely present as circular DNA. In contrast, the low levels of viral genome still present at late times post infection with E6-TTL mutant virus was sensitive to exonuclease indicating that the remaining viral genome was either integrated or compromised otherwise. Upon long term culturing, HFK infected with E6- and E7-TTL mutant virus as well as mock-infected HFK started to senesce approximately 25 to 35 dpi, when they reached the end of their life span. The exact timing varied between different HFK lots used. Wt HPV16-infected HFK, however, continued to grow and express high levels of viral transcripts (Fig 6D). We have cultured these cells for additional 50 days without any sign of senescence, suggesting that they are immortalized. Taken together, these results suggest that neither E6 nor E7 are essential for establishing infection. However, E6 protein is essential for episomal genome maintenance, whereas loss of E7 protein does neither impair genome maintenance nor the viral transcription program in the maintenance stage of infection. However, E7 is absolutely necessary for immortalization of primary HFK under our conditions.

## Discussion

Herein, we describe a novel cell culture system that allows the study of the complete HPV16 life cycle following infectious delivery. Rather than binding virus directly to the cell surface, which has been documented to restrict uptake by primary keratinocytes for unknown reasons [35, 36], we used an ECM-to-cell transfer for infection of primary cells. This approach resulted in efficient uptake of viral genome by the majority of cells. Throughout the development of this infection model we used primary cells grown in the presence or absence of the Rho kinase inhibitor Y-27632 and found no significant difference in infection efficiency. Y-27632 has previously been shown to promote immortalization of primary keratinocytes [37, 38]. Taken together, this suggests that immortalization and/or the use of Y-27632 is not essential for increased infection rates. The model mimics natural infection in that (i) it utilizes pre-binding of virions to the basement membrane equivalent; (ii) only the early but not the late promoter is active in undifferentiated HFK; (iii) early and late promoter are responsive to differentiation triggered by growth in methylcellulose or organotypic raft cultures; (iv) viral genome remains episomal and is amplified upon differentiation; and (v) capsid proteins are expressed in the upper layers of organotypic rafts. At this time, we can only speculate why ECM-to-cell transfer is superior for infecting primary keratinocytes over direct binding to the cell surface. Since our data taken together with previously published observations suggest that the presence of the ECM component LN332 is important for efficient infection [22, 23], we assume that the interaction of the HPV16 capsid with LN332 induces unique conformational changes possibly allowing for direct transfer to the cellular uptake receptor. Indirect evidence for unique contributions of LN332 to HPV16 infection has been presented before using heparan sulfate binding-deficient mutants, which were shown to be non-infectious when bound to the cell surface but fully infectious when pre-bound to ECM in the absence of heparan sulfate moieties [26]. Furthermore, HFK are polarized and uptake via the basolateral surface may be more efficient than uptake by the apical surface. Similar observations have been made for other epitheliotropic viruses [39].

Most models of the HPV life cycle assume that incoming viral genome is amplified, which is followed by subsequent genome maintenance and low transcriptional activity of viral promoters after infections have been established in the basal cell compartment. They also depict early promoter activation when infected keratinocytes enter terminal differentiation, in addition to the well-studied late promoter activation. It is unclear whether genome amplification requires an initial boost of transcription and whether the shift to genome maintenance is accompanied by early promoter repression. Also, no robust experimental data exist in support of viral genome amplification following infectious delivery. The current cell culture models using immortalized cells do not allow studying the temporal regulation of viral promoters during the immediate early stages of the viral life cycle. In addition, the early promoter is only weakly upregulated upon differentiation. We now find that the p97 early promoter strongly responds to differentiation, which, in turn, suggests that the early promoter is repressed in the basal cells. We also found that the splice variant encoding for E8^E2 is the only early transcript whose relative levels increase over time post infection of monolayer cells. E8^E2 is a potent inhibitor of viral replication and transcription and has been shown to restrict viral genome copy numbers in HPV-harboring immortalized cells [28–30]. E8^E2 is transcribed from a recently identified promoter located in the E1 ORF [40]. The E8 promoter has not been studied in great detail, notably, knowledge about its temporal regulation post infectious delivery of viral genome is completely lacking. The infection model will provide a potent platform to study the temporal regulation of the E8 promoter following infectious delivery of viral genome. It is tempting to speculate that its regulation may allow the E8^E2 repressor to orchestrate the shift from establishment of infection, which has been suggested to involve a boost of viral transcription and genome amplification [30], to maintenance transcription and replication.

Despite extensive studies regarding the functions of early viral proteins in immortalization, transformation and transcriptional regulation, we still know very little about their roles during the viral life cycle; owing mainly to our inability to establish cell lines carrying mutations in many viral genes. We generate HPV16 virions in the HEK 293TT cell line, which does not require HPV factors other than the capsid proteins expressed from a heterologous expression vector. Therefore, the system is amenable to extensive mutational manipulation. As a proof of principle that the infection model will allow investigation of the contributions of individual viral proteins to the complete viral life cycle, we tested E1-, E6-, and E7-TTL mutant viruses for their ability to establish infection and retain episomal genome. As expected, the E1-TTL mutant was unable to efficiently establish infection. Viral transcripts are present, however, at levels 1% below that of wt HPV16 at 6 dpi. In turn, this indirectly suggests that viral genome is amplified following infectious entry. However, it is also conceivable that replication is essential for efficient transcription and further experimentation is required to clarify this point. In contrast, E6- and E7-TTL mutant virus established infection, suggesting they are not essential for immediate early events of the viral life cycle. However, viral transcript levels were consistently lower after infection with E6- compared to E7-TTL mutant and wt virus. Analysis of infected cells at subsequent passages suggests that E6-TTL failed to retain episomal viral genome and viral transcripts were not detectable. Published data using mutants of HPV16 and HPV31 are somewhat conflicting. For HPV31, it was shown that both E6 and E7 were required to establish stably transfected cell lines containing episomal viral genome. In contrast, HPV16 genome harboring E7 mutations were episomally maintained in immortalized NIKS keratinocytes [41, 42]. It is interesting to note that previously described E7-mediated changes to the host cell transcriptome, many of which involve S phase genes (for recent reviews see [43, 44]), do not seem to be essential for genome maintenance, as the cells infected with E7-TTL mutant virus retain episomal genomes until they senesce. However, we have not yet compared the host transcripts from cells infected with wild type and E7-TTL mutant virus to formally show which alterations to the transcriptome are seen in wild type-infected cells and which of these are due to E7 expression.

The infection model will provide a unique platform to identify host cell factors transcriptionally regulated by the viral oncoproteins after infectious delivery of viral genome without the requirement for immortalization. Analyses of transcripts isolated from individual layers of the stratified epithelia obtained after growth of infected and immortalized HFK as organotypic raft cultures may provide important clues regarding the involvement of altered pathways in the viral life cycle. In future studies, it should be possible to link alterations of the transcriptome to specific functions of the oncoproteins by using mutant viruses. While many of the biological functions and interacting partners of E6 and E7 are identical between low- and high-risk HPV types, it is still not clear, which activities of the high-risk HPV types are ultimately responsible for immortalization. The infection model should be extendable to the study of low-risk HPV types such as HPV6 and 11, which cannot be studied with the current cell culture systems due to their inability to immortalize keratinocytes. A comparative analysis combined with a genetic approach should identify activities absolutely essential for completion of the viral life cycle of both virus groups and may in turn identify functions mediating immortalization. The low-risk HPV types are known not only to cause genital warts but also recurrent respiratory papillomatosis, a debilitating disease requiring repeated surgical procedures, for which no treatment other than surgery is currently available [45]. The extension of the herein described infection model to low-risk HPV types will provide the first platform to investigate and test potential drug candidates for treatment. The infection model may also allow the investigation of skin cancer-linked HPV types from the β-genus and their cooperation with UV irradiation, including the proposed hit and run mechanism of carcinogenesis [46]. Overall, the establishment of this infection model will provide a new experimental tool for the study of the HPV life cycle and will help further our understanding of the biological processes leading to immortalization. Furthermore, it will be helpful for the emerging field of studying the synergy of different pathogens in the development of tumors such as oropharyngeal squamous cell carcinoma [47, 48].

## Materials and methods

### Cell lines

Human embryonic kidney 293TT and HeLa cells were obtained from John Schiller and Daniel DiMaio, respectively [15, 49]. They were cultured in DMEM supplemented with 10% FBS, non-essential amino acids, antibiotics, and L-Glutamax. Spontaneously immortalized human keratinocytes HaCaT cells were purchased from the American Type Culture Collection (ATCC) and grown in low glucose DMEM containing 5% FBS and antibiotics. Human foreskin keratinocytes (HFKs) were derived from neonatal human foreskin epithelia and maintained in E medium containing mouse epidermal growth factor (EGF) and mitomycin-treated mouse 3T3 J2 fibroblasts as previously described [50]. Pooled primary epithelial keratinocytes were also purchased from the ATCC (PCS-200-010) and used in some experiments. In early experiments where indicated, we maintained and infected primary keratinocytes in the presence of the Rho kinase inhibitor (ROCK) Y-27632, which was reported to increase their lifespan [37, 38]. However, the ROCK inhibitor was excluded prior and during experiments involving long term culturing of infected primary cells. Stable cell lines containing HPV16 episomes were created by co-transfection of pEGFP-N1-HPV16 containing the HPV16 genome (W12 strain) with an expression vector for Cre recombinase and a Neomycin resistance plasmid. Cells were transfected using polyethyleneimine (PEI; Polysciences), selected with G418, and expanded as previously described [51]. Episomal maintenance of the viral genome was confirmed using Southern blotting [51]. Differentiation was induced by suspending cells in 1.5% methylcellulose (MC) for 24 hours followed by washes in phosphate buffered saline [32]. Human primary tonsil cells were isolated from tonsils and maintained in E medium with mitomycin-treated mouse 3T3 J2 fibroblasts. Before harvesting RNA or DNA, fibroblast feeders were removed by short trypsin treatment, followed by two washes in PBS.

### Ethics statement

Foreskin and tonsillar keratinocytes were collected from discarded tissue following routine circumcisions and tonsillectomy from anonymous donors attending University Health, Shreveport. Because the samples were de-identified, would otherwise have been discarded, and were not collected specifically for our studies, the LSUHSC-S IRB ruled that they fell under the NIH’s definition of “exempt” from human subjects research, including informed consent (Institutional IRB approval number: STUDY00000187).

### Generation of HPV16 pseudo- and quasivirions

The pSheLL16 L1/L2 packaging plasmid and pfwB plasmid, expressing enhanced green fluorescent protein (GFP) were a kind gift from John Schiller, Bethesda, MA. The plasmid pEGFP-N1 containing the entire floxed HPV16 genome (pEGFP-N1-HPV16) and pBCre plasmid have been described previously [52]. Quasivirions were generated using 293TT cells following the improved protocol of Buck and Thompson [16] with minor modifications. Briefly, 293TT cells were first cotransfected with the pSheLL16 L1/L2 and pEGFP-N1-HPV16 plasmids and 24 hours later transfected with the pBCre plasmid. An additional two days later, cells were harvested and viral particles were purified as described previously [16, 53]. Because activity of the Cre recombinase generates two circular plasmids of packable size (pEGFPN1 and HPV16 genome), isolated viral particles comprise a mixture of pseudovirions (pEGFPN1 plasmid) and quasivirions (HPV16 genome). Pseudovirions harboring GFP were also generated in 293TT cells as described by Buck et al. [16, 53]. For pseudogenome detection by fluorescence microscopy, pseudogenomes were labeled with EdU (5-ethynyl-2’-deoxyuridine) by supplementing the growth medium with 100 μM EdU at 6 hours post transfection as described [54] during generation of pseudovirions. The viral genome equivalence (vge) was determined by real-time quantitative PCR (RT-qPCR) of encapsidated DNA isolated using the NucleoSpin Blood QuickPure (Macherey-Nagel; 740569.250).

To introduce a TTL into the E1 ORF, the pEGFP-N1-HPV16 plasmid was digested with ApaI for 1 h at 25 C. The subsequent ~4500 bp fragment was excised from the gel, purified using DNA gel clean-up kit (Macherey-Nagel, 740609.50), and subcloned into pBlueScript KS II. Next, we used site-directed mutagenesis to substitute a single nucleotide at position 892 within the E1 ORF, which introduced an in-frame TAA ‘stop’ codon just downstream of the E1 start codon (as previously described by [55]). Once confirming the substitution by DNA sequencing, we re-digested the plasmids with ApaI, excised and gel-purified the mutated fragment and vector, and re-ligated it back into the original pEGFP-N1-HPV16 plasmid. The correct insert was again confirmed by sequencing. Primers used: Forward 5'- CCA TGG CTG ATC CTG CAG GTA CCA ATG GGT AAG AGG GTA CGG GAT GTA ATG G -3', Reverse 5'- CCA TTA CAT CCC GTA CCC TCT TAC CCA TTG GTA CCT GCA GGA TCA GCC ATG G -3'. The E7-TTL mutant has been described previously [51]. Site directed mutagenesis to create TTL mutations in the E6 open reading frame of pEGFP-N1-HPV16 was performed using the QuickChange II Site Directed Mutagenesis kit (Agilent) using primers 5’-GCAATGTTTCAGGACCCATAGTAGTGACCCAGAAAGTTAC-3’ and 5’-GTAACTTTCTGGGTCACTACTATGGGTCCTGAAACATTGC-3’ and confirmed by sequencing.

### Infection using extracellular matrix (ECM)-to-cell transfer

HaCaT cells were seeded in 60 mm cell culture dishes and grown for 24-48h until they reached confluency to allow secretion of ECM. Cells were incubated in Dulbecco’s PBS supplemented with 0.5 mM EDTA for up to 2 h in order to remove the cells. To prevent outgrowth of residual HaCaT cells, the dish surface was treated with 8 μg/ml mitomycin for 4 h. Optiprep-purified viral particles (>5 x 10^7^ vge/dish) diluted in 2 ml E medium were added to the ECM for at least 2h at 37°C. At this time, 5 x 10^5^ low passage primary keratinocytes were added. Two hours later approximately 1 x 10^5^ mitomycin-treated fibroblast feeder cells were added in addition. When different sized culture dishes (ranging from 12 well plates to 100 mm dishes) were used, cell and vge numbers were scaled proportionally to the surface area. Infection was continued for up to 7 days or until cells reached confluency.

### Methylcellulose-induced differentiation of HFK

In order to induce differentiation of HFK cells, cells were suspended in methylcellulose at 5 to 7 days post infection with HPV16 quasivirions as described [56]. Samples were collected 24 or 48 hours later. Increased levels of differentiation markers were confirmed by Western Blot and RT qPCR.

### Organotypic raft cultures

Organotypic raft cultures generated from HFK cells infected for 5 to 7 days with HPV16 quasivirions were grown as described [50, 57]. Briefly one million keratinocytes were seeded onto the surface of the collagen gel containing fibroblasts feeders. Following attachment, the gel with keratinocytes layer was lifted and placed onto a stainless steel grid in a culture dish. Culture medium was added to the dish so that the keratinocyte/collagen plug was exposed to the air from above and to the medium from below. The medium was changed every other day maintaining the air-liquid interface. Rafts were grown for 14 days and samples were collected for RNA/DNA analysis and immunofluorescent staining and FISH. Rafts generated from uninfected HFK seeded on ECM were used as control.

### Immunofluorescence and fluorescent *in situ* hybridization

HFK cells were infected with EdU-labeled pseudovirions using ECM-to-cell transfer on glass slides. EdU staining was performed according to the manufacturer’s directions. In brief, at the indicated times post infection, cells were washed with PBS and fixed with 4% paraformaldehyde for 15 min at room temperature, washed, permeabilized with 0.5% Triton X-100 in PBS for 10 min, washed, and blocked with 5% goat serum in PBS for 30 min followed by a 30 min incubation with Click-iT reaction cocktail containing AlexaFluor 555 for EdU-labeled pesudogenome detection. After extensive washing, cells were incubated for 30 min with anti-PML (BETHYL; A301-167A), and anti-laminin A/C (Sigma; SAB4200236) primary antibodies at room temperature, washed again extensively, and subsequently incubated with AlexaFluor488- and AlexaFluor647-tagged secondary antibodies (Molecular Probes; A11029, A21245) for 30 min. After extensive washing with PBS, cells were mounted in ‘Gold Antifade’ containing DAPI (Life Technologies; P3693).

### Immunofluorescent staining and FISH raft sections

HPV16 genomic DNA probes for FISH were prepared by gel purification of the entire HPV16 genome from pUC-HPV16 digested with *BamHI* and generated using BioNick labeling system according to the manufacturer’s protocol (Invitrogen, 18247–015). When mentioned, raft sections were stained for the presence of viral proteins prior to *in situ* hybridization. Paraffin wax embedded sections were dewaxed in series of xylene and alcohol washes, followed by antigen retrieval using microwave heating at 100°C in citrate buffer with 0.05% Tween for 20 minutes. Slides were permeablized with 0.5%Triton x100 for 45 minutes and blocked with 5% goat serum for 1h. Primary antibodies: anti-L1-7, anti-E1^E4 (a kind gift from J. Doorbar), anti-PCNA (Santa Cruz Biotechnology; sc-7907), anti-p53 (Santa Cruz Biotechnology; sc-126) or anti-MCM7 (Abcam; ab52489) were added for overnight incubation at 4°C. After extensive washing with PBS, sections were incubated for 1h with AlexaFluor-tagged secondary antibodies (Molecular Probes; A21236; A21245; A11030; A11035) for 1 hour. After extensive PBS washing, sections were fixed and slides were treated with 100 ug/ml RNase A in 2× SSC for 1 hour at 37°C and for 5 min with micrococcal nuclease (NEB; M0247S). Enzymatic activity was blocked by adding 20mM EGTA for 5 min. Subsequently, the slides were washed three times with 2× SSC, then dehydrated for 2 min each in 70% EtOH, 80% EtOH and 100% EtOH at room temperature. Slides were then denatured in 70% formamide-2× SSC at 76°C for 3 minutes, followed by dehydration for 2 min each in 70% EtOH (−20°C), 80% EtOH and 5 min in 100% EtOH at room temperature. The probe was denatured at 74°C for 10 minutes prior to hybridization overnight at 37°C. After overnight incubation, the slides were washed multiple times, and tyramide-enhanced fluorescence was carried out according to the manufacturer's instructions (Molecular Probes, T20932). After extensive final washing with PBS, cells were mounted in ‘Gold Antifade’ containing DAPI (Life Technologies; P3693). All IF images were captured by using a Leica CTR6000 fluorescence microscope or by confocal microscopy with a 63x objective using a Leica TCS SP5 Spectral Confocal Microscope and processed with Adobe Photoshop software.

### RNA isolation, cDNA synthesis, real-time qPCR

Total RNA from HFK cells was extracted using the RNeasy Plus Mini RNA Isolation Kit (Qiagen; 74236). RNA samples from raft cultures were extracted using RNA Stat-60 (amsbio LLC) according to manufacturer’s protocol. Isolated RNA samples were treated with DNase I (NEB; M0303L) prior to reverse transcription. 1 or 0.5 μg total RNA was used to reverse-transcribe into cDNA using ImProm-II Reverse Transcriptase kit (Promega). Equal amounts of cDNA were quantified by RT-qPCR using the IQ SYBR Green Supermix (BIO-RAD) and a CFX96 Real-Time System (BIO-RAD). PCR reactions were carried out in triplicate, and transcript levels were normalized to cyclophilin A. Mock reverse-transcribed samples were included as negative control. A list of oligonucleotide sequences used is provided in S2 Table. The BIO-RAD CFX Manager 3.1 software was used to analyze the data.

### RNA sequencing

Total RNA was harvested as described above. RNA quality was assessed on an Agilent Tapestation Bioanalyzer. All samples showed an RNA Integrity Number (RIN) greater than 7. An mRNA sequencing library was prepared with the NEBNextUltra directional library kit and the TruSeq stranded mRNA kit (Illumina). Paired end sequencing (2x75cycles) was performed on an Illumina NextSeq 500 obtaining over 25 million reads per sample. Reads were aligned to the HPV16 (NC_001526.3) genome using STAR_2.4.2a and counted using RSEM 1.2.31.

### Southern Blot

HFK cells were infected with HPV16 quasivirions using ECM to cell transfer. Uninfected cells served as a control. Genomic DNAs (gDNAs) were isolated from the cells cultured in monolayer for 4 days or cultured in monolayer for 4 days followed by 48h in methylcellulose. Cell pellets were resuspended in lysis buffer (400 mM NaCl, 10 mM Tris-HCl [pH 7.4], and 10 mM EDTA); then, RNase A (50 μg/ml), proteinase K (50 μg/ml) and 0.2% SDS were added, and the lysates were incubated overnight at 37°C. DNA was extracted with phenol-chloroform and precipitated with ethanol. Approximately 5 μg of gDNA was digested with *BglII* (which does not cut the HPV16 genome) and resolved on a 0.8% agarose gel. Genomic DNA fragments were transferred from the gel to DuPont GeneScreen*Plus* nylon membrane (NEN Research Products, Boston, Mass.) as described by the manufacturer using alkaline transfer. Prehybridization of the membrane was performed for 1h at 42°C using a solution containing 50% formamide, 4× SSC, 5× Denhardt's solution, 1% SDS, 10% dextran sulfate, and denatured salmon sperm DNA (0.1 mg/ml). The HPV16 probe was prepared by gel purification of the entire HPV16 genome from pUC HPV16 digested with *BamHI* and labeling with the Ready-To-Go DNA labeling kit (Amersham Pharmacia). Labeled probe was then purified with ProbeQuant G-50 Micro columns (Amersham Pharmacia), denatured, and added to fresh hybridization solution, which was incubated with membrane at 42°C overnight. Membrane was washed twice with 2× SSC-0.1% SDS for 15 min at room temperature, twice with 0.5× SSC-0.1% SDS for 15 min at room temperature, twice with 0.1× SSC-0.1% SDS for 15 min at room temperature, and once with 0.1× SSC-1% SDS for 30 min at 50°C. Hybridizing species were visualized by autoradiography.

### Western blot

Whole-cell extracts were obtained from cell pellets lysed in 1x Laemmli Sample Buffer (BIO-RAD) supplemented with 2-mercaptoethanol. Proteins were resolved on SDS-PAGE and transferred to nitrocellulose membranes (BIO-RAD). Membranes were blocked 1 hour in 5% Blotting-Grade Blocker (BIO-RAD) in 1× TBST) and incubated at 4°C overnight with anti-cytokeratin 10 (Santa Cruz Biotechnology; sc-52318), anti-E7 (Santa Cruz Biotechnology, sc-6981) or anti-b-actin (Santa Cruz Biotechnology; sc-47778) primary antibodies. After incubation, membranes were washed 3 × 15 minutes in 1× TBST wash buffer. Membranes were then incubated with horseradish peroxidase–tagged goat anti-mouse and goat anti-rabbit secondary antibodies (1:2500, Jackson ImmunoResearch) at room temperature for 1 hour, washed 3 × 15 minutes in 1× TBST. Signals were detected by enhanced chemiluminescence (Thermo Scientific). Equal protein loading was confirmed by probing with β-actin monoclonal antibody.

### OncoE6 cervical test

The presence of E6 protein in infected cells was detected using a kit from ArborVita according to the manufacturer’s protocol. Briefly, the cell lysate was incubated with alkaline phosphatase conjugated high-affinity E6 HPV16/18 monoclonal antibodies. Next, a nitrocellulose test strip with two capture lines consisting of immobilized mAbs to HPV16/18 E6 was placed into the lysate/mAb-AP mix. The solution was allowed to migrate through the strip by capillary action. E6-mAb-AP present in the sample is forming a ternary complex with the immobilized antibodies on the strip. The complex was visualized as a purple line in the respective location on the strip by the addition of an enzyme substrate solution provided in the kit.

### Viral genome resistance to exonuclease 5

Genomic DNA was isolated using the QIAamp DNA Blood Mini Kit (Qiagen) according to the manufacturer’s instructions and stored at 4°C. DNA from UMSCC47 and HPV16-infected 293TT cells served as an HPV16 integration control and episomal HPV16 control, respectively. 100 ng of DNA was either treated with exonuclease V (RecBCD, NEB) or left untreated for 1 hour at 37°C followed by heat inactivation at 95°C for 10 minutes. 10 ng of digested/undigested DNA was then quantified by real time PCR using a 7500 FAST Applied Biosystems thermocycler with SYBR Green PCR Master Mix (Applied Biosystems) and 300nM of each primer in a 15 μl reaction. Nuclease free water was used in place of the template for a negative control. The following cycling conditions were used: 50°C for 2 minutes, 95°C for 10 minutes, 40 cycles at 95°C for 15 seconds, and a dissociation stage of 95°C for 15 seconds, 60°C for 1 minute, 95°C for 15 seconds, and 60°C for 15 seconds. Separate PCR reactions were performed to amplify HPV16 E6 (1) (F: 5’-GAGAACTGCAATGTTTCAGGACC-3’ R: 5’-TGTATAGTTGTTTGCAGCTCTGTGC-3’), human mitochondrial DNA (2) (F: 5’-CAGGAGTAGGAGAGAGGGAGGTAAG-3’ R: 5’-TACCCATCATAATCGGAGGCTTTGG -3’), and human 18S_ribosomal DNA (3) (F: 5’-GCAATTATTCCCCATGAACG-3’ R: 5’-GGGACTTAATCAACGCAAGC-3’). Human mitochondrial DNA and 18S ribosomal DNA served as episomal and multi-copy linear DNA internal controls, respectively. Primer efficiencies were based on a standard curve generated using a 5-fold dilution series of undigested UMSCC47 DNA and used to calculate the relative amount of DNA per sample. The percent of DNA resistant to exonuclease digestion was calculated relative to undigested DNA.

## Supporting information

S1 Fig **(A)** E1^E4 transcripts from HPV16-infected HFK (maintained in the presence of 10 μM Y027632) isolated at 2, 4, 7 and 10 dpi were quantified by qRT-PCR. HFK were either left on virus-loaded ECM for the entire time period (HFK) or reseeded after 2 dpi onto virus-free ECM (HFK reseeded). **(B)** ECM depositions generated by HaCaT, HeLa or HFK cells were used for ECM-to-cell transfer. RNA was isolated at 3 dpi. Note that HeLa ECM does not support efficient ECM-to-cell transfer, which is most probably due to a lack of LN332 expression.
(TIF)

S2 Fig Quantification of viral transcripts isolated from human tonsillar epithelial cells (HTE) at 7 dpi with HPV16. (TIF)

S1 Table Relative levels of viral splice variants. (TIF)

S2 Table List of oligonucleotides used in the study. (TIF)
