# Supplementary figures and images for: A new cell culture model to genetically dissect the complete human papillomavirus life cycle
Source: PLoS Pathog. 2018 Mar 1;14(3):e1006846. doi: 10.1371/journal.ppat.1006846 (PMC5833277; doi:10.1371/journal.ppat.1006846)

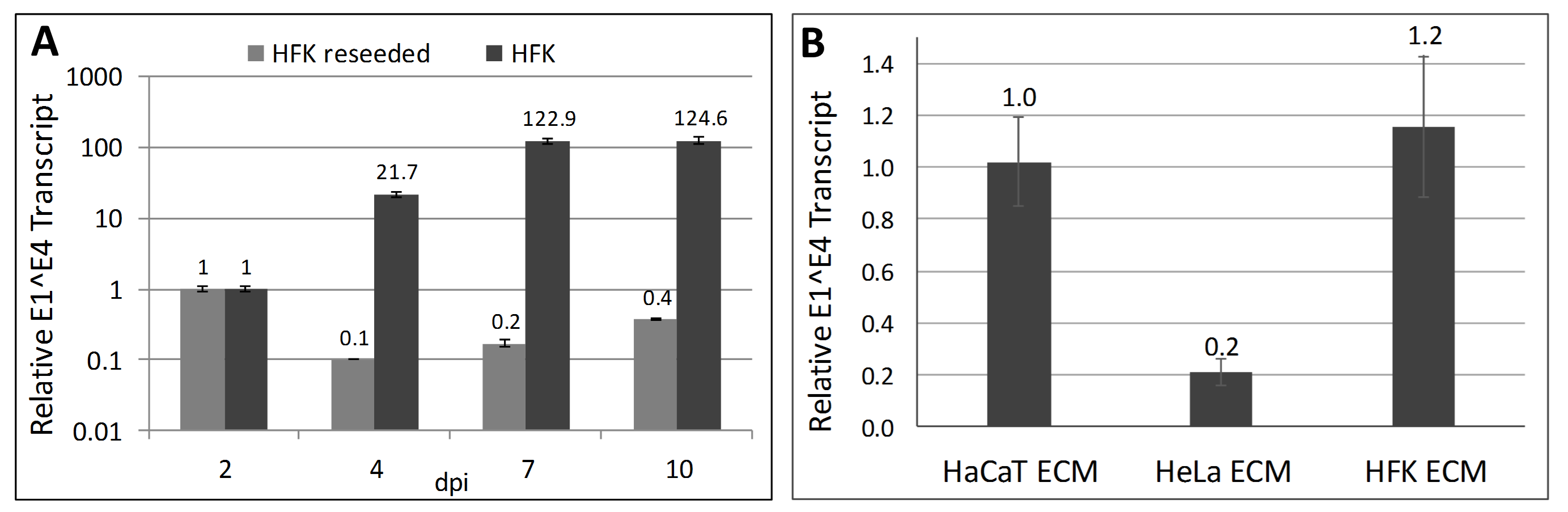

Supplement: S1 Fig — (A) E1^E4 transcripts from HPV16-infected HFK (maintained in the presence of 10 μM Y027632) isolated at 2, 4, 7 and 10 dpi were quantified by qRT-PCR. HFK were either left on virus-loaded ECM for the entire time period (HFK) or reseeded after 2 dpi onto virus-free ECM (HFK reseeded). (B) ECM depositions generated by HaCaT, HeLa or HFK cells were used for ECM-to-cell transfer. RNA was isolated at 3 dpi. Note that HeLa ECM does not support efficient ECM-to-cell transfer, which is most probably due to a lack of LN332 expression. (TIF) [file ppat.1006846.s001.tif]

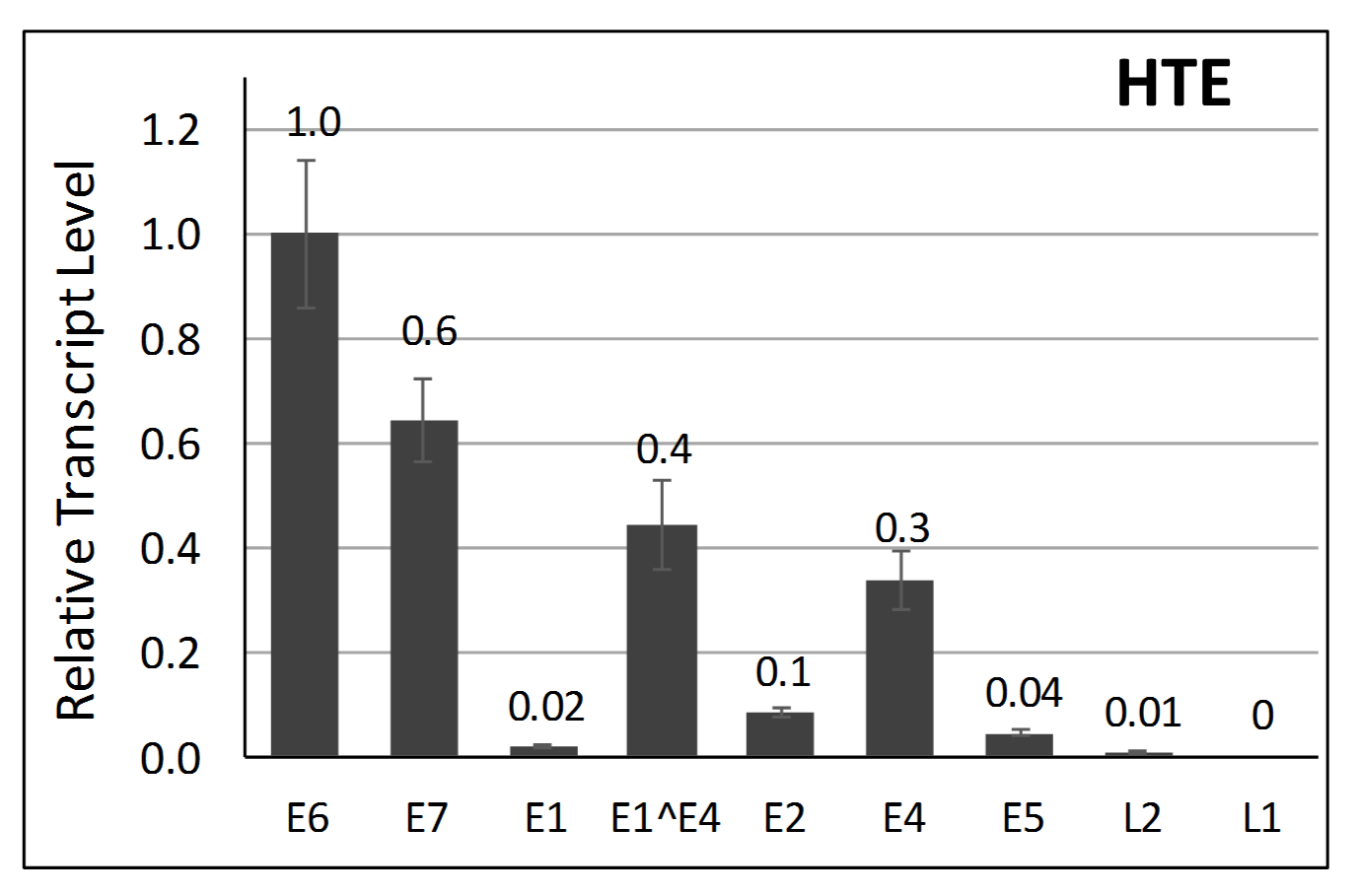

Supplement: S2 Fig — (TIF) [file ppat.1006846.s002.tif]

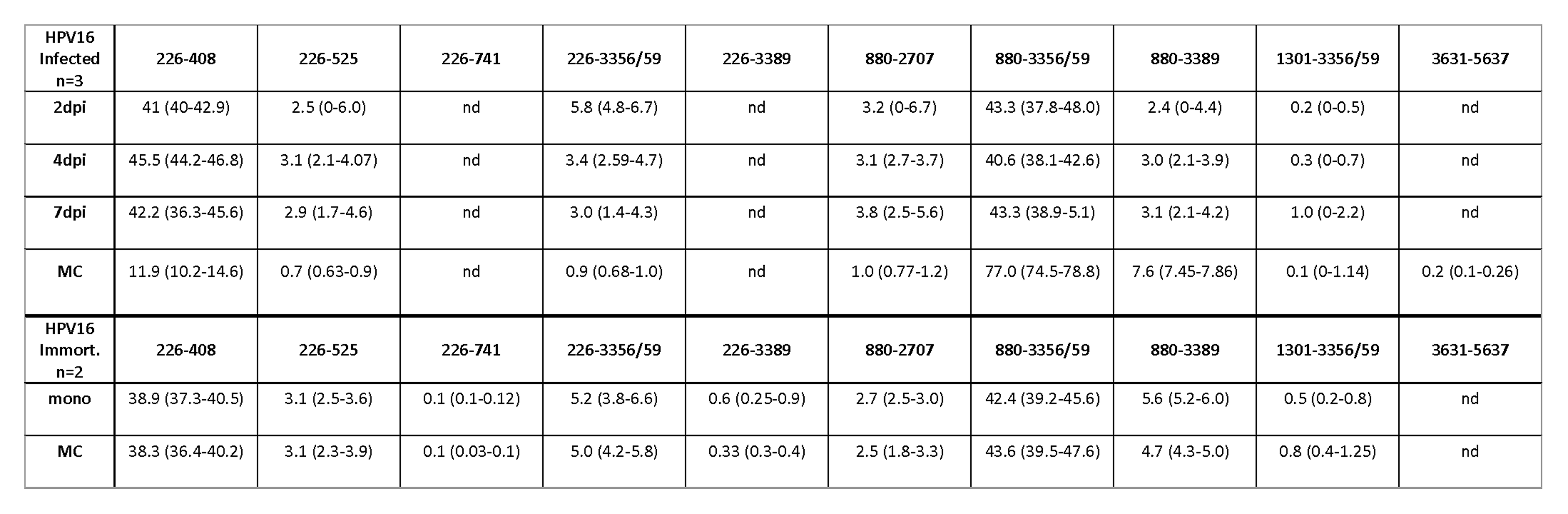

Supplement: S1 Table — (TIF) [file ppat.1006846.s003.tif]
